# Controlling hydrogel properties by tuning non-covalent interactions in a charge complementary multicomponent system†

**DOI:** 10.1039/d1sc02854e

**Published:** 2021-07-22

**Authors:** Santanu Panja, Annela Seddon, Dave J. Adams

**Affiliations:** School of Chemistry, University of Glasgow Glasgow G12 8QQ UK dave.adams@glasgow.ac.uk; School of Physics, HH Wills Physics Laboratory, University of Bristol Tyndall Avenue Bristol BS8 1TL UK; Bristol Centre for Functional Nanomaterials, HH Wills Physics Laboratory, University of Bristol Tyndall Avenue Bristol BS8 1TL UK

## Abstract

Mixing small molecule gelators is a promising route to prepare useful and exciting materials that cannot be accessed from any of the individual components. Here, we describe pH-triggered hydrogelation by mixing of two non-gelling amphiphiles. The intermolecular interactions among the molecules can be tuned either by controlling the degree of ionization of the components or by a preparative pathway, which enables us to control material properties such as gel strength, gel stiffness, thermal stability, and an unusual shrinking/swelling behaviour.

## Abstract 2

The properties of a charge complementary multicomponent gel can be tuned either by pH change or by varying the preparative pathway.

## Graphical abstract

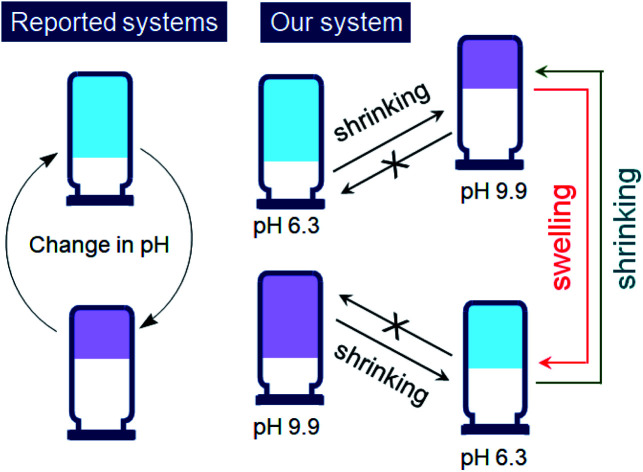

## Introduction

Supramolecular gels are useful in many areas including optoelectronics and biomedical applications.^1–3^ Such gels result from the self-assembly of small molecules in solution under the influence of various non-covalent interactions such as hydrogen bonding, π-stacking, hydrophobic interactions and ionic interactions.^4,5^ As the intermolecular interactions are individually weak and reversible, tuning of the gel properties is possible by altering the nature of the intermolecular forces on applying stimuli like pH, temperature, UV-light, ionic analytes *etc.*^6–8^ Generally, supramolecular gels are obtained from single components. However, the mixing of two different molecules in preparing gels is a promising way of accessing useful and exciting materials that cannot be derived from either of the individual components.^9–12^ Adaptation of gel properties is further possible by selective modulation of the individual components which extends the number of applications.^10,12,13^ When two different compounds are mixed, broadly two types of gels are possible depending on the self-assembly pattern. A self-sorted gel can be formed where individual fibre formation occurs from each gelator; both types of fibres then contribute to form the matrix.^14^ Alternatively, the gel fibres may be formed from both the components resulting in cooperative assembly or co-assembly.^14^

A well-known method of synthesizing multicomponent gels is to mix two organic compounds bearing pH responsive, oppositely charged functionalities.^10,11,15–20^ In these gels, the electrostatic interactions between the positively and negatively charged groups act as the key driving force to build up the underlying network.^12,20–23^ Additionally, tuning of material properties is possible simply by controlling the surface charge on the fibres through pH change.^22^ Many natural systems and processes such as assembly of proteins and silk fibrils involve multicomponent ionic interactions.^24^ Recently, the Stupp group utilized electrostatic interactions between two pH-responsive charged peptides to achieve hierarchical assembly formation of proteins.^25^ There is a common tendency to prepare multicomponent gels where both or at least one of the components inherently has the ability to form a gel.^11,15,26–31^ As one example, the Smith group used ionic interactions between carboxylic acid and amine groups to modify hydrogel properties by chiral selection within gels.^23^ In comparison, mixing of two non-gelling compounds resulting in the formation of a gel is unusual and challenging.^11,20,32–35^ For instance, in a recent study, Hu *et al.* demonstrated two co-assembled peptide systems that aggregate to form gels involving electrostatic interactions although none of the components self-assembled into hydrogels by themselves.^36^

**Fig. 1:**
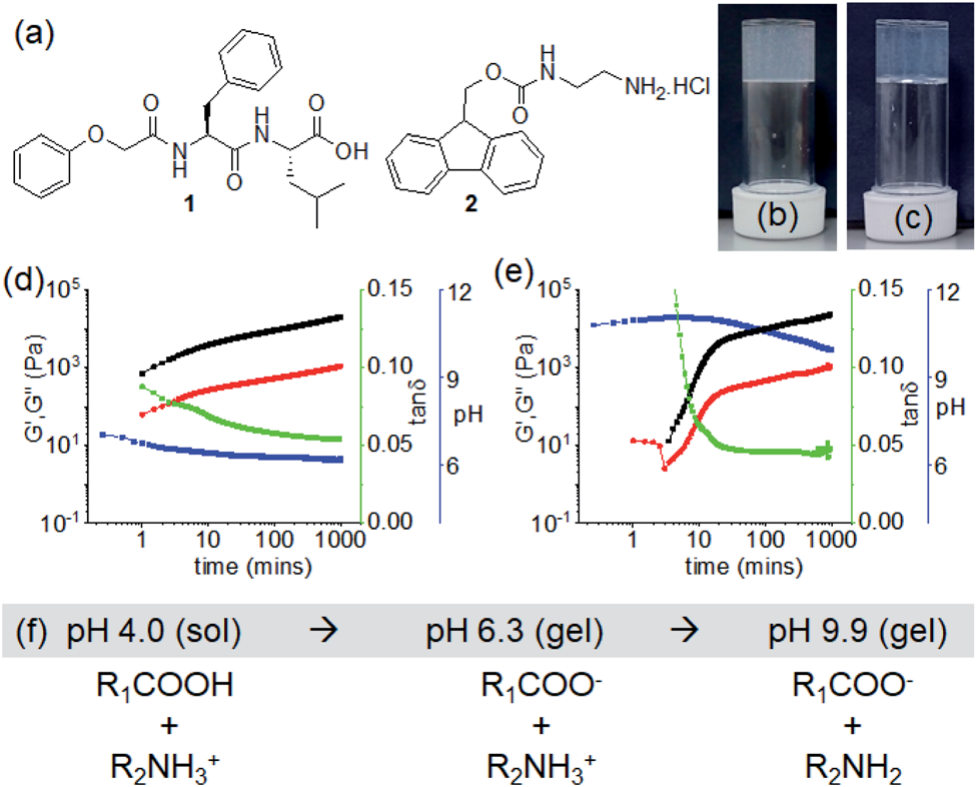
(a) Chemical structures of compounds **1** and **2**. (b and c) Photographs of the hydrogels of (**1** + **2**) obtained at pH 6.3 and pH 9.9 respectively. (d and e) Variation of pH (blue), G′ (black), G′′ (red) and tan *δ* (green) with time for the mixture of **1** and **2** in presence of 0.005 M (d) and 0.011 M (e) of NaOH. The final pH is 6.3 (d) and 9.9 (e). (f) Changes in chemical structures of **1** (R_1_COOH) and **2** (R_2_NH_3_^+^) on gradual increase of pH in solution. For (b–e), in all cases, initial concentrations of **1** and **2** are 2 mg mL^−1^ (2 mg mL^−1^ of each in the multicomponent gel), solvent is DMSO/H_2_O (20/80, v/v).

In this work, we report an unusual pH responsive multicomponent gel network by varying the preparative pathway. We describe pH-triggered hydrogelation on mixing of two non-gelling amphiphiles (compounds **1** and **2**) bearing opposite ionizable functionalities (Fig. 1a). The solubility of the molecules in solution can be controlled by the degree of ionization of the functional groups. Sequential deprotonation of **1** and **2** using a base enables us to control the intermolecular interactions resulting in different co-assembled gels. We observe that the intermolecular interactions among the molecules are highly dependent on the preparative pathway. Pathway dependence is common in supramolecular gels.^37,38^ The gel properties can vary depending on the method used to perform the gelation even though the starting and final conditions of the materials are the same. In our study, a kinetic barrier is encountered when interconversion between different gel states is achieved by a post-gelation pH change (a post assembly fabrication (PAF)) leading to gels with improved mechanical properties. We also find that on successive pH change, the resulting PAF gels exhibit reversible shrinking/swelling behaviour, which is very uncommon for such supramolecular gels.

## Results and discussion

To form a gel, a balance between the hydrophobicity and hydrophilicity of the amphiphiles is required that drives the fibre formation and gelation. Dipeptide **1** alone formed a precipitate with a pH of 4.2 when a DMSO solution is diluted with water (DMSO/H_2_O, 20/80, v/v) (Fig. S1†). At a pH higher than the apparent p*K*_a_ of **1** (*ca.*5.4, Fig. S2†), a free-flowing solution was obtained (pH 7.1 with equimolar amounts of NaOH) (Fig. S1†). The more hydrophilic compound **2** (apparent p*K*_a_ is 8.6, Fig. S2†)^31^ remained in solution both at acidic and basic pH (pH are 5.3 and 9.6 in absence and presence of equimolar amounts of NaOH respectively) (Fig. S1†). Hence, while present as single components, both **1** and **2** behave as non-gelling components either at acidic or basic pH in DMSO/H_2_O (20/80, v/v). At both sets of conditions, the small angle X-ray scattering (SAXS) data for **1** and **2** is weak in intensity, implying a lack of significant self-assembled structure (Fig. S3†). Simple mixing of DMSO solution of **1** and aqueous solution of **2** resulted in precipitation with a pH of 4.0 (Fig. S4†).

We then targeted successive deprotonation of **1** and **2** by adding different concentrations of NaOH so as to control the degree of ionization of each amphiphile. The amounts of NaOH required to sequentially deprotonate **1** and **2** were calculated from the molar concentrations of the individual components. At a concentration of 0.005 M of NaOH, only the terminal carboxylic acid of **1** (0.0048 M) undergoes deprotonation while compound **2** (0.0062 M) exists in its ammonium form. Increasing the concentration of NaOH to 0.011 M (equal to the sum of molar concentrations of **1** and **2**) resulted in deprotonation of both **1** and **2.** Interestingly, we obtained a gel under both conditions with a pH of around 6.3 and 9.9 respectively (Fig. 1b and c). The gel at pH 9.9 was translucent as compared to a more turbid gel at pH 6.3. The SAXS data for the two gel states show that the scattering intensity is higher than for the precursor solutions (Fig. S3†). However, at the concentrations used here, the data are difficult to fit.

The non-covalent interactions in the co-assembled gels significantly depend on the degree of ionizations of **1** and **2**, and also influence the gelation kinetics. Initially, time sweep rheology was conducted to probe the development of the gels. For the low pH gel (pH 6.3), gelation begins immediately after mixing of solutions of **1** and **2** in presence of 0.005 M of NaOH as observed by rheology with the storage modulus (G′) significantly greater than the loss modulus (G′′) at initial times (Fig. 1d and S5†). A self-supporting gel was formed which exhibited no flow on inversion of the vial after 5 minutes (Fig. S6†). The rheological moduli evolve with time and reach a plateau after 3 hours. In comparison, the hydrogelation of **1** and **2** at pH 9.9 showed a different behaviour. Increasing NaOH concentration to 0.011 M resulted in a considerable delay in the appearance of gel (Fig. 1e, S5 and S6†). Gelation begins after 3 minutes with the appearance of G′ > G′′. Over time, both the G′ and G′′ increased and the tan *δ* (G′′/G′) reached a plateau after almost 5 hours.

**Fig. 2:**
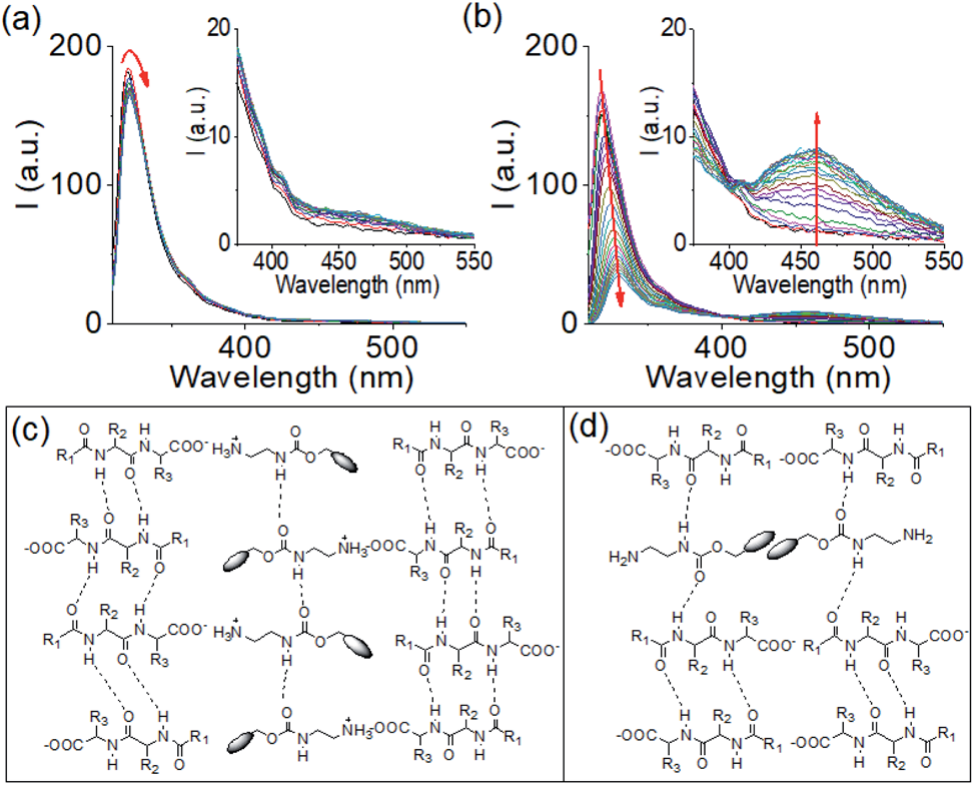
Time variable emission spectra for the mixture of **1** and **2** in presence of 0.005 M (a) and 0.011 M (b) of NaOH. Insets represent expanded section of the corresponding graph. Time variable data for the gels were recorded after 1 min, 2 min, 5 min, 10 min, 15 min, 30 min, 1 h, 1.5 h, 2 h and then after each hour onward until 16 h of addition of the components. The red arrow shows how the spectrum changes with time. (c and d) Probable modes of interaction between **1** and **2** in the multicomponent gel formed at pH 6.3 (c) and pH 9.9 (d). For (c and d): R_1_ = PhOCH_2_, R_2_ = PhCH_2_, R_3_ = Me_2_CHCH_2_ and the blob represents the fluorenyl group. For (a and b), initial concentrations of **1** and **2** are 2 mg mL^−1^, solvent is DMSO/H_2_O (20/80, v/v).

To understand the gelation mechanism, time variable emission spectra of the mixtures of **1** and **2** were collected. **1** is weakly emissive while solution of **2** exhibited a strong monomer emission at 318 nm (Fig. S7†).^31,39^ During multicomponent hydrogelation at pH 6.3, the emission of **2** remained unaffected and experienced only a 3 nm red shift with time (Fig. 1f, 2a, S7 and S8†). At the higher NaOH concentration, the monomer emission of **2** at 318 nm progressively decreased in intensity and red shifted to 330 nm with appearance of a new band in the region 410–525 nm (Fig. 1f, 2b, S7 and S8†).

The spectral changes showed ratiometric response with an isosemissive point at 402 nm (Fig. 2b). The emergence of the new peak at 462 nm validates overlapping of the fluorenyl groups at high pH.^20,31,39,40^ To confirm this, concentration variable emission spectra of the individual components were recorded. Compound **1** exhibited weak emission in the region 350–500 nm (as broad band) both at acidic and basic pH (Fig. S9†). Interestingly, the intensity of the band at both acidic and basic pH decreases with increasing concentration of **1**. In case of compound **2**, the emission in the region 410–525 nm was observed only at high pH (Fig. S10†). A dilution in the concentration of **2** at high pH resulted in a decrease in the intensity of the band at 410–525 nm region and corroborates the excimer formation due to overlapping of the fluorenyl groups (Fig. S9†).^31,39,41^ Furthermore, comparison of emission profiles of the mixture of **1** and **2** at different pH revealed that no excimer formation occurred either when both the compounds present in their original forms (protonated) or when **1** undergoes deprotonation but **2** exists as ammonium cation (Fig. S8†). The excimer peak due to aromatic stacking between the Fmoc-moieties appears only when compound **2** undergoes deprotonation at high pH (Fig. S8†). These results show that, comparing the multicomponent gels at pH 6.3 and 9.9, aromatic stacking between the Fmoc-groups exists only at pH 9.9 (Fig. S8†). Moreover, increasing NaOH concentration resulted in a red shift of around 2 nm in the absorption spectra of the gel (Fig. S11†). From FTIR spectroscopy, the stretching signal for the carboxylic carbonyl of **1** which appears at 1723 cm^−1^ in its amorphous state, disappears in the multicomponent gel. Instead, a broad signal appears at 1593 cm^−1^ confirming the formation of the carboxylate ion (Fig. S12†).^31^ In the gels, the carbamate –CO stretching of **2** appeared at 1693 cm^−1^ compared to 1687 cm^−1^ in amorphous state. The sharp signals for the amide –NHs and carboxylic-OH of **1** merged with the –NH stretching of **2** and moved to the lower region with significant broadening due to intermolecular hydrogen bonding in the multicomponent gels. In the gels, the stretching signals near 1674 cm^−1^ and 1650 cm^−1^ suggest formation of antiparallel β-sheet-like structures through intermolecular hydrogen bonding involving the amide carbonyls of **1**.^42,43^ Interestingly, the stretching frequency of the amide carbonyls of **1** at 1645 cm^−1^ in the gel at pH 6.3 shifted to 1643 cm^−1^ with increase in pH indicating existence of stronger hydrogen bonded network in the gel formed at pH 9.9. We suggest that, at pH 6.3, the electrostatic interactions between the negatively charged carboxylate and positively charged ammonium groups allows faster gelation (Fig. 2c and S6†). Increasing the concentration of NaOH resulted in deprotonation of both **1** and **2** and thereby diminished the electrostatic interactions due to loss of charge on amine form of **2**. However, formation of hydrophobic amine of **2** enables maintaining the overall hydrophobic/hydrophilic balance and so trigger co-assembly involving other non-covalent interactions like hydrogen bonding, stacking *etc* (Fig. 2d). The degree of different non-covalent interactions result in different rates of gelation. To get more insight, gelation studies were conducted by varying the concentrations of the components. From Fig. S13,† it is evident that salt formation involving the electrostatic interactions between the carboxylate and ammonium groups of **1** and **2** respectively, resulted in gelation in all combinations whereas no gel formation occurred when both the components underwent deprotonation at high pH. The extent of aromatic stacking (*i.e.*, the intensity of the excimer bands) at high pH decreases either on reduction of total concentration of **2** or on diminution of relative concentration of **2** (Fig. S8 and S13†). These results endorse that while the electrostatic interactions acted as the key driving force to form a gel at low pH, aromatic stacking was the major contributor to drive gelation at high pH. Moreover, at a particular concentration of **1** and **2**, the electrostatic interactions were more effective in bringing gelation than the aromatic stacking (Fig. S13†).

**Fig. 3:**
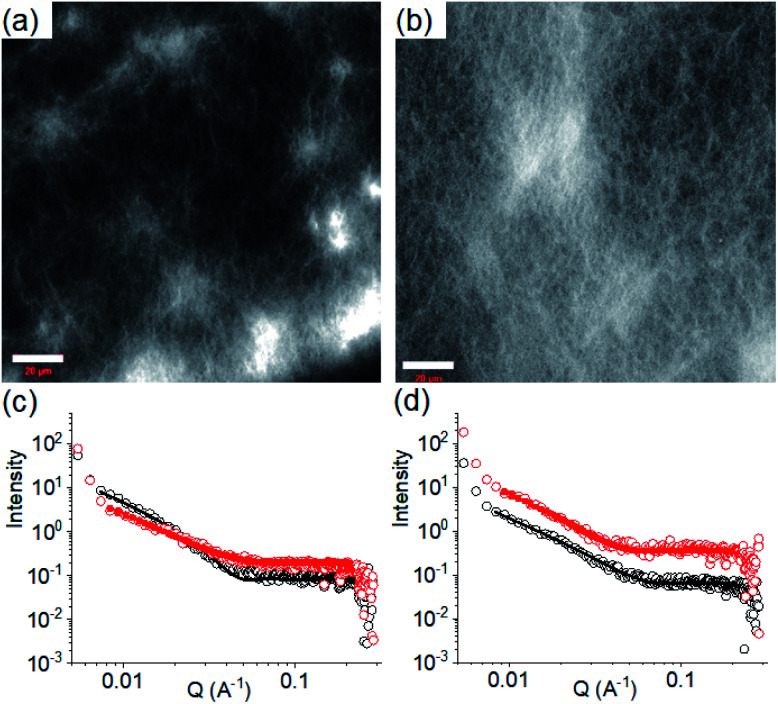
Confocal fluorescence microscopy images (scale bars represent 20 μm) of the multicomponent gel of **1** and **2** obtained at pH 6.3 (a) and pH 9.9 (b). (c) Shows SAXS data and fits for systems obtained by PAF. (d) Shows SAXS data and fits for systems obtained after a pH cycle. For (c) and (d), the black data are for the multicomponent gels at pH 9.9 and the red data for the multicomponent gels at pH 6.3. The open symbols show the data and the solid lines the fits to the data as discussed in the text. The error bars for the SAXS data are omitted here for clarity but are shown in Fig. S3.† In all cases, initial concentrations of **1** and **2** are 2 mg mL^−1^, solvent is DMSO/H_2_O (20/80, v/v).

Interestingly, whilst the SAXS data are weak (Fig. S3†), the scattering patterns are very similar for both gels. This implies that the underlying self-assembled structures are similar. There are differences on longer length scales, *i.e.*, the microstructure of the gels that can be seen by confocal microscopy (Fig. 3). Note that drying of such gels usually results in morphological changes^44^ and so confocal microscopy was used to allow imaging in the gel state. Interestingly, while the gel at 6.3 contained more spherulitic domains, the high pH gel exhibited a higher density of long fibres. The differences in the microstructures of the gels can be ascribed to the rate of gelation (Fig. 2d,e and S6†) which influences the growth of fibres as well as their distribution and crosslinking across a larger length scale.^37^ Different microstructures result in variations in the bulk properties of the materials which was evident from rheological studies. The low pH gel showed higher stiffness (G′) compared to the gel at pH 9.9 although no significant change in gel strength (the strain at which the gel breaks) was found (Fig. S14†). In addition, the thermal stability (*t*_gel_) of the gels decreased from 50 °C to 30 °C on increasing the pH from 6.3 to 9.9 (Fig. S14†). While the low pH gel exhibited complete recovery of both G′ and G′′ after a heat–cool cycle, a significant decrease of rheological moduli was found for the high pH gel. The higher stiffness as well as better thermal stability of the gel at pH 6.3 was due to the existence of stronger intermolecular interactions than the gel at pH 9.9 (as evident from Fig. S13†). Hence, despite similar underlying self-assembled structures, the differences in the microstructure translate directly into differences in the mechanical properties of the gels.

**Fig. 4:**
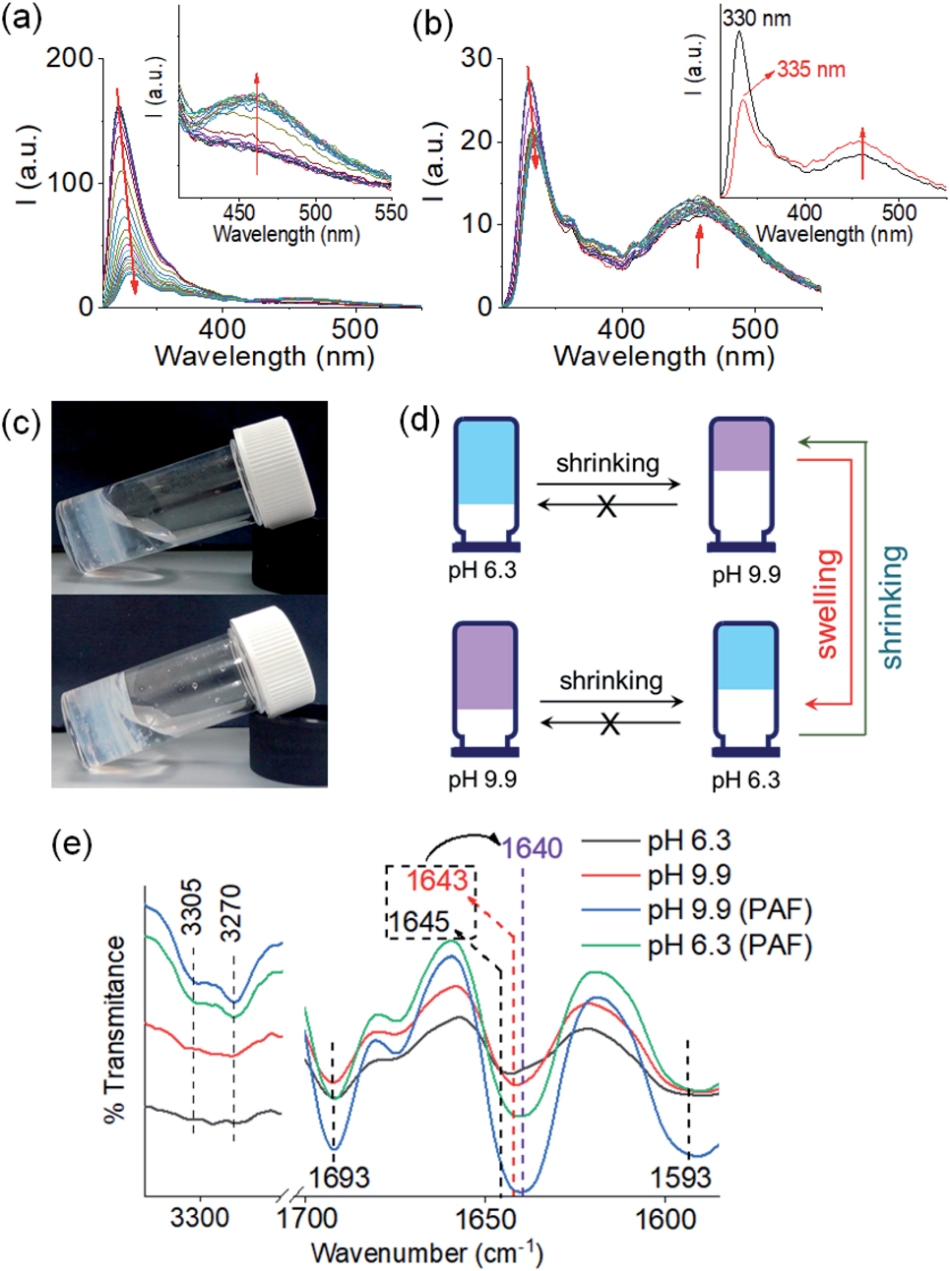
(a) Time variable changes in emission of the multicomponent gel of **1** and **2** at pH 6.3 (a) and pH 9.9 (b) upon addition of 0.006 M of NaOH and 0.006 M of HCl respectively. Time variable data for the gels were recorded after 1 min, 2 min, 5 min, 10 min, 15 min, 30 min, 1 h, 1.5 h, 2 h and then after each hour onward until 16 h of addition of the components. Inset of (a) represents expanded section of the graph. Inset of (b) represent the emission spectra of the multicomponent gel of **1** and **2** before (black) and after (red) addition of 0.006 M of HCl at pH 9.9. The red arrow shows how the spectrum changes with time. (c) Photographs of the hydrogels of (**1** + **2**) obtained at pH 9.9 (top) and pH 6.3 (bottom) involving post assembly pH change. (d) Cartoon representing pathway dependent changes in volume of gels obtained from the mixtures of **1** and **2** at different pH. (e) Partial FTIR spectra of the hydrogels of (**1** + **2**) at different pH prepared by different methods. In all cases, initial concentrations of **1** and **2** are 2 mg mL^−1^, solvent is DMSO/H_2_O (20/80, v/v).

We next applied a PAF method to achieve a transition between these two pH dependent gel states. When 0.006 M of NaOH was added on the top of the gel at pH 6.3 to target the final pH 9.9 (*i.e.*, the total NaOH concentration becomes 0.011 M), slow diffusion of NaOH into the gel resulted in gradual increase of both G′ and G′′ over time (Fig. S15†). A concomitant reduction in the value of tan *δ* further corroborates an increase in the solid-like nature of the gel. Following the process using fluorescence spectroscopy revealed that the emission of the gel at 321 nm gradually red shifted by 11 nm (Fig. 4a). The emergence of the excimer peak at 462 nm confirms formation of a new type of molecular packing with the pH increase. Using UV-vis spectroscopy, the peak at 300 nm became broad as the pH increased from 6.3 to 9.9 (Fig. S16†). Interestingly, there is a significant change in the SAXS data (Fig. 3 and S3†); the scattering intensity increases significantly, and the data can now be fitted to a flexible cylinder model with a radius of around 7 nm (Table S1†). There is therefore a change in packing leading to a change in the self-assembled structures when using a PAF method as compared to direct formation of the gel at the same pH. This shows that the structures present are due to the process by which they are formed as opposed to be down the absolute pH.

Similarly, addition of 0.006 M of HCl onto a preformed gel at pH 9.9 caused gradual decrease of pH to 6.3 (total NaOH concentration becomes 0.005 M) (Fig. S15†). Again, the gel evolved to a stiffer material (higher G′) compared to the initial gel. UV-vis studies showed that there was an increase in intensity at the region 270–280 nm (Fig. S16†). Again, there are differences in the SAXS for the gels formed by PAF and those formed directly at pH 6.3 (Fig. 3 and S3†), implying that there is again a change in the structures when PAF is used to bring about a pH decrease. Whilst the data are weak for the directly prepared gel, the scattering is stronger for the gel formed by PAF and can again now be best fit to a flexible cylinder model with a radius of 5.2 nm. In the absorption spectrum, the peak at 302 nm was also blue shifted slightly to 300 nm. Surprisingly, by fluorescence, the emission of the gel at 330 nm further red shifted to 335 nm with reduced intensity (Fig. 4b). Unlike the gel obtained directly at pH 6.3, the gel prepared by PAF at same pH exhibited fluorenyl excimer emission at 462 nm indicating that aromatic stacking acts as an additional driving force in maintaining the hydrogel network (Fig. S17†). Compared to the directly-prepared gels at both pH 6.3 and pH 9.9, PAF method leads to better overlapping of the aromatic Fmoc groups with an increase in intensity of the excimer band at 462 nm (Fig. S17†). These results demonstrate a kinetic barrier that does not simply allow reversible modification of the intermolecular interactions, but drives the systems to another state where aromatic stacking plays an decisive role in maintaining the co-assembled network with other non-covalent forces. The results again reveal that at a particular pH, the intermolecular interactions among the molecules depend on preparative pathway.

The gels obtained through post assembly pH change exhibit unusual shrinking in gel volume (Fig. 4c and d). The volume of the gel reduced by ∼30% on increasing pH from 6.3 to 9.9. When HCl was used to reduce the pH from 9.9 to 6.3, the resulting low pH gel showed approximately ∼21% contraction in volume compared to the initial high pH gel. Shrinkage, or syneresis, of supramolecular gels is unusual.^45–47^ Normally, such syneresis is limited to cross-linked polymer gels. Here, the syneresis can be ascribed to the pH induced change in molecular packing (as observed from UV-vis and fluorescence) that allows the molecules to stack effectively (Fig. S17†) and establish stronger intermolecular interactions (Fig. S18†).^47^ From FTIR spectroscopy, 3–5 cm^−1^ decrease of the amide stretches of **1** show formation of a stronger hydrogen bonded network and support our proposition (Fig. 4e). Consequently, structural changes were observed in the gel network upon syneresis. An evolution to a more densely packed network of long fibres occurred in both cases (Fig. S19†).

**Fig. 5:**
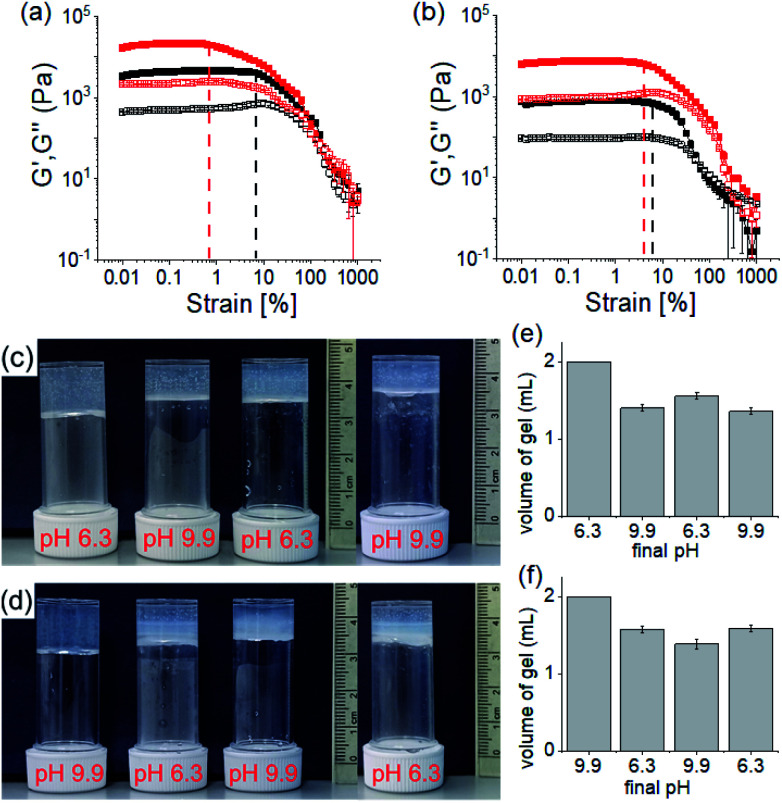
Strain sweep experiments of the hydrogels of (**1** + **2**) at pH 6.3 (a) and pH 9.9 (b) prepared directly (black) or by PAF method (red). The closed symbols represent G′, the open symbols G′′. The dotted vertical lines represent the maximum strain bearing capacity of the corresponding hydrogels. (c and d) Photographs showing the changes in gel volume on successive pH change starting from a directly prepared gel at pH 6.3 (c) and 9.9 (d). (e) and (f) are the bar graphs representing the final volume of the respective gels obtained from (c) and (d) respectively. In all cases, initial concentrations of **1** and **2** are 2 mg mL^−1^, solvent is DMSO/H_2_O (20/80, v/v).

The rheological data shows that, at a particular pH, the gels obtained through PAF exhibited ≥5 times increase in robustness of the materials as compared to those formed directly at the same pH (Fig. 5a, b and S20†). The stiffer gels showed lower strain bearing capacity. The thermal stability of the gels was also improved by 8–10 °C (Fig. S12 and S21†). However, irrespective of method of preparation, the low pH gels exhibited complete recovery of both G′ and G′′ after a heat–cool cycle whilst the high pH gels did not.

We further adjusted the pH by adding 0.006 M of NaOH and HCl (as suitable depending on the system) to investigate if the PAF gels exhibit swelling (Fig. 4d, 5c and d). A further change in pH of the PAF gel from pH 9.9 to 6.3 resulted in ∼11% increase in gel volume (Fig. 5c). Changing the pH from 6.3 to 9.9 caused further ∼12% shrinking of the PAF gel (Fig. 5d). The SAXS data show that the structures present are similar to those formed by the first PAF. In both cases, a flexible cylinder model best fits the data. At pH 6.3, the model implies that the structures have a radius of around 6.1 nm, similar to that formed initially on PAF at pH 9.9 (Table S1†). For the cycle finishing at pH 9.9, the radius is around 4.8 nm, close to that initially formed by the PAF at 6.3. Hence, it appears that the further pH cycle results in little change in the underlying structures formed during the PAF (Fig. 3 and S3†), implying that these volume changes must be driven by changes in microstructure rather than changes in the primary self-assembled structures. Initial observation of the Kuhn length of each sample shows that the PAF prepared at pH 6.3 has a longer Kuhn length (and thus less flexible gel fibres) than the other samples. On cycling this sample to pH 9.9, the Kuhn length drops significantly. This may be due to subtle changes in the fibre structure due to the fibres become more hydrophobic at higher pH, leading to increased flexibility, however care should be taken in interpreting this data due to the relatively large errors on the fit of the Kuhn length. Interestingly, at a particular pH, the final volume of the resulting gels (after a pH cycle) was almost equal to the volume of the gels obtained by PAF method described in Fig. 4 (Fig. 5c–f). Moreover, at a particular pH, the gels also exhibit similar rheological properties (Fig. S22†) and a similar microstructure (Fig. S19 and S23†). Surprisingly, unlike the directly prepared gels, the gels obtained by PAF at both pH 6.3 and 9.9 exhibit reversible swelling/deswelling on successive pH change (Fig. 5c–f) presumably driven by a change in hydrophobicity of the fibres.^46^ We suggest that formation of the amine in the gel matrix at pH 9.9 resulted in increase in hydrophobicity of the fibres which instigate expelling water from the hydrogel network and the gel shrunk.^46^ Changing pH to 6.3 enables regeneration of hydrophilic ammonium cation and so the water molecules enters into the fibrous network allowing gel swelling.^46^ Hence, we were not only able to control the mechanical properties but also the volume of gels by varying the pH and preparative pathway.

## Conclusions

In conclusion, we have demonstrated pH-triggered multicomponent hydrogelation involving two non-gelling components bearing opposite ionizable functionalities. Sequential deprotonation of the compounds using a base led to different co-assembled gels where either the electrostatic interactions or the aromatic stacking prevailed predominantly and stabilized the hydrogen bonded network. The intermolecular interactions can also be tuned by varying the preparative pathway. Our system encountered a kinetic barrier that imposes syneresis when interconversion between the gel states was carried out by post assembly pH change (PAF). The syneresis occurred due to rearrangement in molecular packing that enables the molecules to stack effectively and establish stronger intermolecular interactions. The shrunken gels exhibit higher rigidity compared to the gels prepared directly. Interestingly, the gels obtained by PAF (*i.e.*, the shrunken gels) exhibits reversible swelling/deswelling behaviour on successive pH change due to the alteration of hydrophobicity of the fibres.

pH driven shrinking/swelling of charge complementary multicomponent gels are rare.^46^ Generally, the directly prepared gels exhibit reversible swelling/shrinking behaviour.^45–49^ One potential advantage of our designed system is that the directly prepared gel first allow accessing a second gel state with improved rigidity upon pH change (Fig. 4d). This modified gel then exhibits reversible swelling/deswelling on further alteration of pH (Fig. 4d). Hence, we were not only able to control the mechanical properties but also the spatial programming of gels (volume of gels) by varying the pH and preparative pathway.

## Data availability

We have provided all the necessary data in the ESI.

## Author contributions

Conceptualisation (SP, DA); data collection (SP, AS); Data analysis (all); funding acquisition (AS, DA); methodology (all); project administration (DA); supervision (DA); writing (all).

## Conflicts of interest

There are no conflicts to declare.

## Supplementary Material

SC-012-D1SC02854E-s001

## References

[cit1] Du X., Zhou J., Shi J., Xu B. (2015). Chem. Rev..

[cit2] Deen G. R., Loh X. J. (2018). Gels.

[cit3] Hoque J., Sangaj N., Varghese S. (2019). Macromol. Biosci..

[cit4] Terech P., Weiss R. G. (1997). Chem. Rev..

[cit5] Amabilino D. B., Smith D. K., Steed J. W. (2017). Chem. Soc. Rev..

[cit6] Panja S., Adams D. J. (2021). Chem. Soc. Rev..

[cit7] Zhang W., Gao C. (2017). J. Mater. Chem. A.

[cit8] Echeverria C., Fernandes S. N., Godinho M. H., Borges J. P., Soares P. I. P. (2018). Gels.

[cit9] Buerkle L. E., Rowan S. J. (2012). Chem. Soc. Rev..

[cit10] Okesola B. O., Mata A. (2018). Chem. Soc. Rev..

[cit11] Li L., Sun R., Zheng R. (2021). Mater. Des..

[cit12] Raymond D. M., Nilsson B. L. (2018). Chem. Soc. Rev..

[cit13] Lau H. K., Kiick K. L. (2015). Biomacromolecules.

[cit14] Draper E. R., Adams D. J. (2018). Chem. Soc. Rev..

[cit15] Roy K., Chetia M., Sarkar A. K., Chatterjee S. (2020). RSC Adv..

[cit16] Gayen K., Nandi N., Das K. S., Hermida-Merino D., Hamley I. W., Banerjee A. (2020). Soft Matter.

[cit17] Soto Morales B., Liu R., Olguin J., Ziegler A. M., Herrera S. M., Backer-Kelley K. L., Kelley K. L., Hudalla G. A. (2021). Biomater. Sci..

[cit18] Tena-Solsona M., Alonso-de Castro S., Miravet J. F., Escuder B. (2014). J. Mater. Chem. B.

[cit19] Redondo-Gómez C., Abdouni Y., Becer C. R., Mata A. (2019). Biomacromolecules.

[cit20] Criado-Gonzalez M., Wagner D., Rodon Fores J., Blanck C., Schmutz M., Chaumont A., Rabineau M., Schlenoff J. B., Fleith G., Combet J., Schaaf P., Jierry L., Boulmedais F. (2020). Chem. Mater..

[cit21] Toksoz S., Mammadov R., Tekinay A. B., Guler M. O. (2011). J. Colloid Interface Sci..

[cit22] Seroski D. T., Dong X., Wong K. M., Liu R., Shao Q., Paravastu A. K., Hall C. K., Hudalla G. A. (2020). Commun. Chem..

[cit23] Edwards W., Smith D. K. (2014). J. Am. Chem. Soc..

[cit24] Nakamura H. (2009). Q. Rev. Biophys..

[cit25] Wester J. R., Lewis J. A., Freeman R., Sai H., Palmer L. C., Henrich S. E., Stupp S. I. (2020). J. Am. Chem. Soc..

[cit26] Vieira V. M. P., Hay L. L., Smith D. K. (2017). Chem. Sci..

[cit27] Saddik A. A., Chakravarthy R. D., Mohammed M., Lin H.-C. (2020). Soft Matter.

[cit28] Wang F., Feng C.-L. (2018). Chem. - Eur. J..

[cit29] Okesola B. O., Wu Y., Derkus B., Gani S., Wu D., Knani D., Smith D. K., Adams D. J., Mata A. (2019). Chem. Mater..

[cit30] Su T., Hong K. H., Zhang W., Li F., Li Q., Yu F., Luo G., Gao H., He Y.-P. (2017). Soft Matter.

[cit31] Panja S., Shebanova O., Smith A., Dietrich B., Adams D. J. (2021). Angew. Chem., Int. Ed..

[cit32] Aboudzadeh M. A., Muñoz M. E., Santamaría A., Fernández-Berridi M. J., Irusta L., Mecerreyes D. (2012). Macromolecules.

[cit33] Pal A., Basit H., Sen S., Aswal V. K., Bhattacharya S. (2009). J. Mater. Chem..

[cit34] Zhang F., Xu Z., Dong S., Feng L., Song A., Tung C.-H., Hao J. (2014). Soft Matter.

[cit35] Liu Y., Wang T., Liu M. (2012). Chem. - Eur. J..

[cit36] Hu T., Zhang Z., Euston S. R., Geng M., Pan S. (2020). Biomacromolecules.

[cit37] Raeburn J., Zamith Cardoso A., Adams D. J. (2013). Chem. Soc. Rev..

[cit38] Panettieri S., Ulijn R. V. (2018). Curr. Opin. Struct. Biol..

[cit39] Hirst A. R., Roy S., Arora M., Das A. K., Hodson N., Murray P., Marshall S., Javid N., Sefcik J., Boekhoven J., van Esch J. H., Santabarbara S., Hunt N. T., Ulijn R. V. (2010). Nat. Chem..

[cit40] Channon K. J., Devlin G. L., Magennis S. W., Finlayson C. E., Tickler A. K., Silva C., MacPhee C. E. (2008). J. Am. Chem. Soc..

[cit41] Fleming S., Debnath S., Frederix P. W. J. M., Tuttle T., Ulijn R. V. (2013). Chem. Commun..

[cit42] Pelton J. T., McLean L. R. (2000). Anal. Biochem..

[cit43] Chen L., Morris K., Laybourn A., Elias D., Hicks M. R., Rodger A., Serpell L., Adams D. J. (2010). Langmuir.

[cit44] Mears L. L. E., Draper E. R., Castilla A. M., Su H., Zhuola, Dietrich B., Nolan M. C., Smith G. N., Doutch J., Rogers S., Akhtar R., Cui H., Adams D. J. (2017). Biomacromolecules.

[cit45] Xie F., Qin L., Liu M. (2016). Chem. Commun..

[cit46] Zhou S.-L., Matsumoto S., Tian H.-D., Yamane H., Ojida A., Kiyonaka S., Hamachi I. (2005). Chem. - Eur. J..

[cit47] Das B. K., Pramanik B., Chowdhuri S., Scherman O. A., Das D. (2020). Chem. Commun..

[cit48] Wu J., Yi T., Zou Y., Xia Q., Shu T., Liu F., Yang Y., Li F., Chen Z., Zhou Z., Huang C. (2009). J. Mater. Chem..

[cit49] Farahani A. D., Martin A. D., Iranmanesh H., Bhadbhade M. M., Beves J. E., Thordarson P. (2019). ChemPhysChem.

