# Supplementary material for: Controlling hydrogel properties by tuning non-covalent interactions in a charge complementary multicomponent system
Source: Chem Sci. 2021 Jul 22;12(33):11197–203. doi: 10.1039/d1sc02854e (PMC8386653; doi:10.1039/d1sc02854e)
Supplement: SC-012-D1SC02854E-s001 [file SC-012-D1SC02854E-s001.pdf]

Electronic Supplementary Material (ESI) for Chemical Science.
This journal is © The Royal Society of Chemistry 2021

Controlling hydrogel properties by tuning non-covalent interactions in a charge complementary multicomponent system

Santanu Panja,[a] Annela Seddon[b,c] and Dave J. Adams[a,*]

[a] *School of Chemistry, University of Glasgow, Glasgow, G12 8QQ, U.K.*

Email: dave.adams@glasgow.ac.uk

[b] *School of Physics, HH Wills Physics Laboratory, Tyndall Avenue, University of Bristol, Bristol, BS8 1TL, U.K.*

[c] *Bristol Centre for Functional Nanomaterials, HH Wills Physics Laboratory, Tyndall Avenue, University of Bristol, Bristol, BS8 1TL, U.K.*

Supporting Information

Table of Contents

Experimental Details	S3
Supplementary Figures	S6
References	S16

Experimental details

Materials: Compound **1** was synthesised as described previously.[1] Compound **2** was purchased from Sigma Aldrich and used as received. All other chemicals and solvents used were purchased from commercial suppliers and used as received. Deionised water was used throughout all experiments.

Preparation of solutions: Stock solution of **1** was prepared in DMSO at concentrations of 10 mg/mL by stirring. A stock solution of **2** was prepared at a concentration of 4 mg/mL in water by stirring. Solutions of both the compoounds were prepared freshly before each experiment. Stock solutions of NaOH and HCl were prepared at a concentration of 0.1 M in H_2O.

Preparation of gels: The multicomponent hydrogels were prepared from a mixture of **1** and **2** in DMSO/H_2O (20/80 v/v) under different conditions. In preparing gels, initial concentrations of **1** and **2** was maintained as 2 mg/mL (2 mg/mL of each in the multicomponent gels) in all cases. Hence in the gels, the molar concentrations of **1** and **2** were 0.0048 M and 0.0062 M, respectively. The amounts of NaOH require to sequentially deprotonate **1** and **2** was calculated from the molar concentrations of the individual components. We used 0.005 M of NaOH to selectively deprotonate compound **1** while further addition of 0.006 M of NaOH leads to the deprotonation of compound **2** as well. Hence, in the multicomponent gels, the concentration of NaOH was either 0.005 M (equal to molar concentration of **1**) or 0.011 M (equal to the sum of molar concentrations of **1** and **2**). The pH of the gels was measured to be pH (6.3 ± 0.1) and pH (9.9 ± 0.1) in presence of 0.005 M and 0.011 M of NaOH, respectively. To achieve transitions between these two pH dependent gel states, either 0.006 M of NaOH (in case of gels at pH 6.3) or 0.006 M of HCl (in case of gels at pH 9.9) was added onto the pre-formed gels to target the desired final pH. Note that, a shrinking in gel volume occurred when the original gels were subjected to post-assembly pH change (post assembly fabrication, PAF). The resultant PAF gels showed swelling/deswelling on reversal of the pH further. Hence, the PAF gels were directly used without removing the excess solvent in the next step.

To prepare the gels directly at pH 6.3, a mixture of 0.40 mL of the solution of **1** and 0.1 mL of solution of NaOH was prepared in a 7 mL Sterilin vial. To this solution, a mixture of 0.5.0 mL of H_2O and 1.0 mL of solution of **2** was added in one aliquot. To prepare the multicomponent gels at pH 9.9, a mixture of 0.380 mL of H_2O and 1.0 mL of solution of **2** was transferred to the vial containing a mixture of 0.4 mL of **1** and 0.220 mL of NaOH. Therefore, in the respective gels, the initial concentrations of **1** and **2** were 2 mg/mL and concentration of NaOH was 0.005 M or 0.011 M. In both cases, the ratio of DMSO and water was 20:80. Throughout the manuscript, these gels are described as directly prepared gels.

To prepare gels involving post-assembly pH change (post assembly fabrication, PAF), initially gels were prepared at pH 6.3 and 9.9 by following the same methodology as described above. To prepare gels at pH (9.9 ± 0.2) using PAF, 0.120 mL of NaOH was added on the top of the gel prepared at pH 6.3. This makes total NaOH concentration to 0.011 M. Similarly, addition of 0.120 mL of 0.1 M of HCl into the gel at pH 9.9 reduced the total NaOH concentration to 0.005 M and allows to prepare gels at pH (6.3 ± 0.2) by PAF. Throughout the manuscript, these gels are described as gels obtained by PAF method.

We also prepared gels by altering the pH further. To execute this, initially gels were prepared at pH 9.9 and 6.3 involving the PAF method described above, onto which either 0.120 mL of HCl or 0.120 mL of NaOH was added. This leads to complete a pH cycle where the pH of the gels reverts to the initial pH (6.3 ± 0.2) and pH (9.9 ± 0.2), respectively. Apparently, these gels are also prepared by post assembly pH change. However, for simplicity, these gels are described as gels obtained after a pH cycle throughout the manuscript.

pH measurements: A FC200 pH probe from HANNA instruments with a 6 mm x 10 mm conical tip was used for pH measurements. The stated accuracy of the pH measurements is ±0.1. For the gels involving compounds **1** and **2**, the reaction mixtures were prepared as described above at a 2 mL volume in a 7 mL Sterilin vial and the pH change was monitored with time. The temperature was maintained at 25 °C during the measurement by using a circulating water bath.

pK_a determination was carried out by recording the pH values after each addition of HCl (0.1M) to the individual solution of **1** and **2** (concentration is 2 mg/mL) containing 1 molar equivalents of NaOH (0.1 M) in 20% DMSO in H_2O. During the titrations, the solutions were stirred continuously. The experimental temperature was 25 °C.

Rheological measurements: All rheological measurements were undertaken on an Anton Paar Physica MCR 301 and MCR 101 rheometer at 25 °C. Strain, frequency and time sweeps were performed using a vane and cup geometry. Strain sweeps were performed at 10 rad/s from 0.01 % to 1000 % strain. Frequency sweeps were carried out from 1 rad/s to 100 rad/s at 0.5 % strain. All gels were left ~16 hours before being measured. Time sweeps were performed at an angular frequency of 10 rad/s and with a strain of 0.5%. For all experiments, gels were prepared as mentioned earlier in 2 mL volume in a 7 mL Sterilin vials.

For the temperature sweeps, gels were prepared as mentioned earlier in metal rheology cups and left overnight before measurements were carried out. G′ and G″ were recorded at a strain of 0.5% and a frequency of 10 rad/s within the temperature range of 25-80 °C. The heating and cooling rate was 1 °C/min.

Confocal microscopy: A Zeiss LSM710 confocal microscope (Zeiss, Gottingen, Germany) with an LD EC Epiplan NEUFLUAR 50X, 0.55 DIC (Carl Zeiss, White Plains, NY, USA) objective was used for imaging. All gel samples were prepared in presence of Nile blue (2 µL/mL of a 0.1 wt % solution). For the gels obtained directly at pH 6.3 and 9.3, samples were prepared as mentioned earlier in CELLview culture dishes (35 mm diameter). For the gels obtained by PAF, samples were prepared in 7 mL Sterilin vials keeping the same volumes of the components as mentioned earlier. Then small amounts of the gels were deposited onto glass microscope slides. A cover slip was placed carefully on the gel before imaging. All the samples were excited at 633 nm using a He-Ne laser. Images were captured using Carl Zeiss ZEN 2011 v7.0.3.286 software.

UV-Vis measurements: Absorption spectra of **1** and **2** under different conditions were recorded on an Agilent Technologies Cary 60 UV-Vis spectrophotometer using a 0.01 mm path length quartz cuvette. All gel samples were prepared in Sterilin vials using the same methodology as described before and were left overnight. Then, small amounts of the gels were transferred to the cuvette for measurement.

Fluorescence spectroscopy: Data were collected on an Agilent Technologies Cary Eclipse fluorescence spectrometer. Samples were prepared in PMMA cuvettes with a path length of 1 cm by following the same procedure as mentioned before. Time variable data for the gels were recorded after 1 min, 2 mins, 5 mins, 10 mins, 15 mins, 30 mins, 1 h, 1.5 h, 2 h and then after each hour onward until 16 h of addition of the components. Compound **1** produced precipitation (sol state) in absence of base. For such sols, the components were mixed well in solution using a micropipette before recording of the spectra. In all cases, the excitation wavelength was 300 nm. Both the excitation and emission slit widths were 5 nm.

FTIR spectroscopy: Data were recorded using an Agilent Cary 630 FTIR spectrometer (with ATR attachment). For the solid samples (amorphous), the background of the empty ATR crystal was taken. For gels, 20% DMSO in H_2O was used for the background correction. All the gels were prepared following the same methodology as described above. Then, small amounts of the gels were deposited on the ATR crystal before recording the spectra.

Small Angle X-ray Scattering (SAXS): SAXS data were collected on a Ganesha 300XL instrument (Xenocs). Samples were loaded into 3.5mm borosilicate glass capillaries (Capillary Tube Supplies Ltd) and sealed using optical adhesive (Norland) for 30 minutes under UV light. SAXS data were collected at room temperature over a Q range of 0.007 – 0.25$Å^{-1}$ for an exposure time of 3600 seconds. All

measurements were corrected for transmission and absolute intensity and had the solvent background and empty capillary scattering subtracted before processing. Data were reduced using SAXSGUI, and model fits were performed using SASView 4.0.[2]

Supplementary Figures

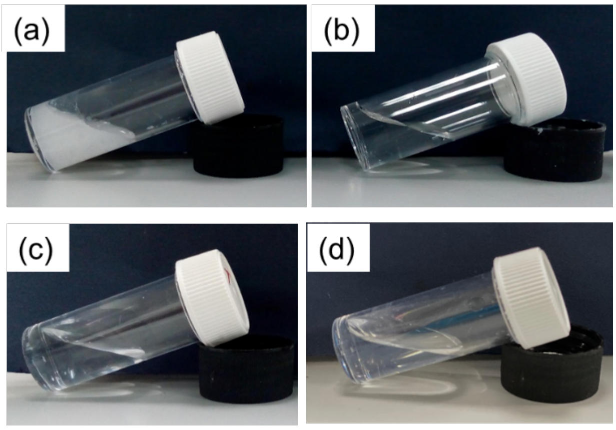

Figure S1. Photographs showing the phase change of **1** (a, b) and **2** (c, d) in absence (a, c) and presence (b, d) of equimolar amounts of NaOH in 20:80 DMSO/water (v/v). In all cases, concentrations of **1** and **2** are 2 mg/mL. The pH of the solutions is (a) 4.2, (b) 7.1, (c) 5.3 and (d).9.6 respectively.

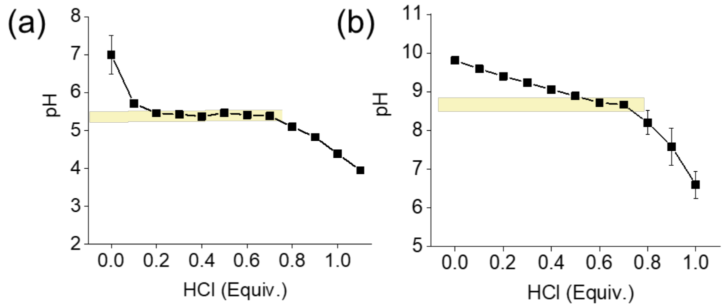

Figure S2. Determination of apparent pK_a of (a) **1** (2 mg/mL) and (b) **2** (2 mg/mL) in 20:80 DMSO/water (v/v). The plateau is taken to represent the apparent pK_a value, shown by the horizontal shading.

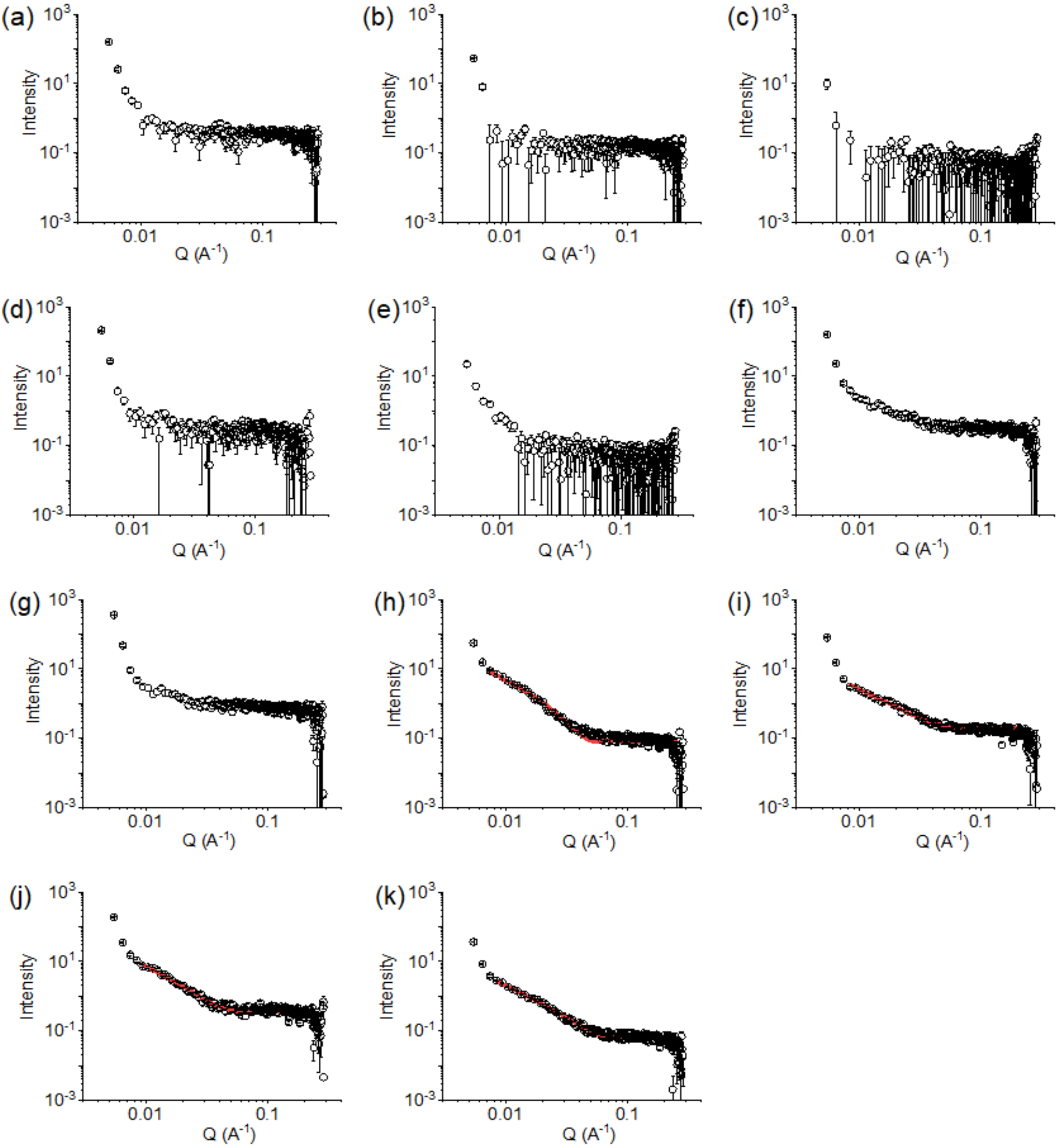

Figure S3. (a-d) SAXS data for **1** (a, c) and **2** (b, d) in absence (a, b) and presence (c, d) of equimolar amounts of NaOH. For (a-d) the pH of the solutions is 4.2, 5.3, 7.1 and.9.6, respectively. (e) SAXS data for the mixture of **1** and **2** (pH 4.0). (f, g) SAXS data for the multicomponent gels of **1** and **2** prepared directly at pH 6.3 and 9.9, respectively. (h, i) SAXS data for for the multicomponent gels at pH 9.9 and 6.3 respectively, obtained by PAF. (j, k) SAXS data for for the multicomponent gels at pH 6.3 and 9.9 respectively, obtained after a pH cycle. In all cases, the open circles show the data. For (h-k), the red lines are the fits to the data. In all cases, concentrations of **1** and **2** are 2 mg/mL, solvent is 20:80 DMSO/water (v/v).

	pH 9.9 (PAF)	pH 6.3 (PAF)	pH 6.3 (Cycle)	pH 9.9 (Cycle)
scale	0.00017421 ± 3.4e-5	0.00015397 ± 9.8e-6	0.00023702 ± 1.2e-5	0.00012526 ± 3.3e-5
background	0.079634 ± 0.0007	0.16433 ± 0.001	0.35742 ± 0.0003	0.064143 ± 0.0006
Length (Å)	>1000	>1000	>1000	>1000
Kuhn length (Å)	114.2 ± 45.3	204.6 ± 15.2	107.7 ± 23.2	82.8 ± 14.3
Radius (Å)	69.5 ± 3.1	51.6 ± 1.44	61.0 ± 2.1	45.9 ± 3.5
Chi^2	7.26	2.78	3.14	1.76

Table S1. Fitting parameters for fits to SAXS data for the multicomponent gels of **1** and **2** at pH 6.3 and 9.9 obtained by PAF method and after a pH cycle.

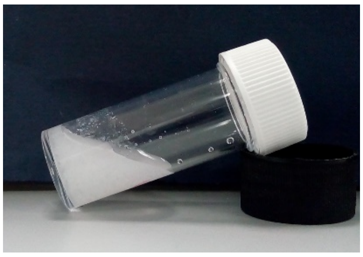

Figure S4. Photograph of the mixture of **1** and **2** in 20:80 DMSO/water (v/v). Concentrations of **1** and **2** are 2 mg/mL. The pH of the solution is 4.0.

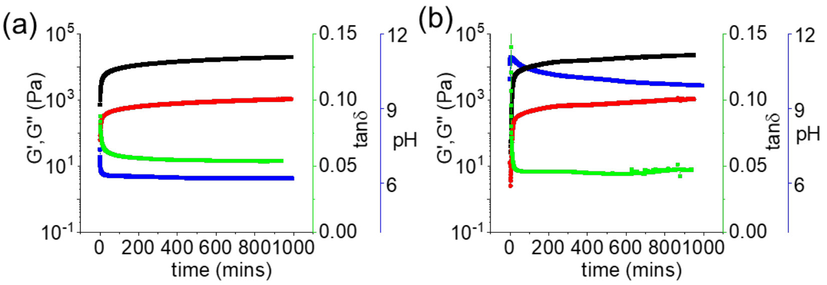

Figure S5. Variation of pH (blue), G′ (black), G″ (red) and tanδ (green) with time for the mixture of **1** and **2** in presence of (a) 0.005 M and (b) 0.011 M of NaOH. Figure S4a and Figure S4b represent Figure 1d and Figure 1e of the manuscript using a linear x-axis scale. In both cases, initial concentrations of **1** and **2** are 2 mg/mL, solvent is DMSO/H_2O (20/80, v/v). The final pH is (a) 6.3 and (b) 9.9.

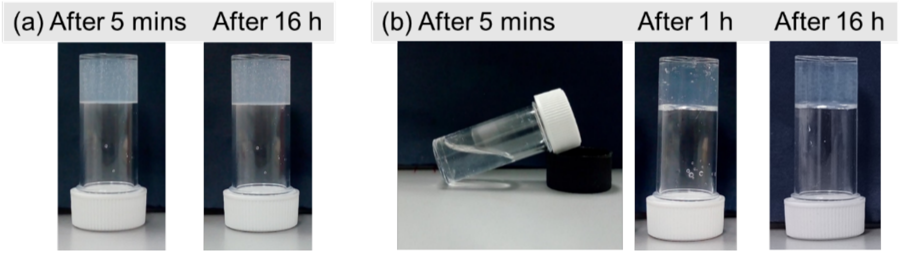

Figure S6. Photographs showing the phase change of the mixture of **1** and **2** with time in presence of (a) 0.005 M and (b) 0.011 M of NaOH. In both cases, initial concentrations of **1** and **2** are 2 mg/mL, solvent is DMSO/H_2O (20/80, v/v). The final pH is (a) 6.3 and (b) 9.9.

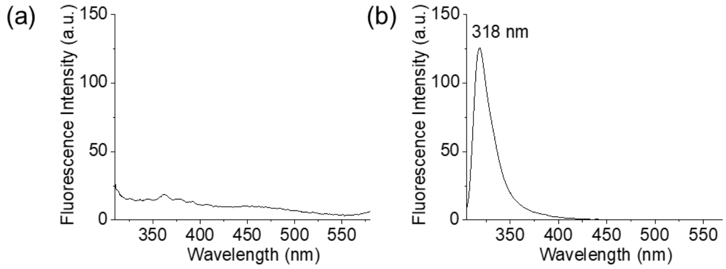

Figure S7. Emission spectra of (a) **1** and (b) **2** in DMSO/H_2O (20/80, v/v). Concentrations of **1** and **2** are 2 mg/mL. The pH of the solutions is (a) 4.2 and (b) 5.3 . The excitation wavelength is 300 nm.

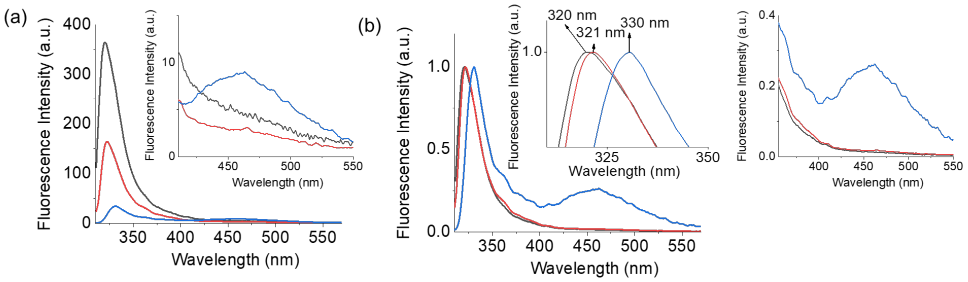

Figure S8. (a) Emission spectra of the sol obtained from the mixture of **1** and **2** (black), and the multicomponent gels of **1** and **2** obtained in presence of 0.005 M (red) and 0.011 M (blue) of NaOH. The final pH is 4.0 (black), 6.3 (red) and 9.9 (blue). Figure (b) is the normalized graph of (a). Insets show an expanded section of the corresponding graph. In all cases, initial concentrations of **1** and **2** are 2 mg/mL, solvent is 20:80 DMSO/water (v/v). The excitation wavelength is 300 nm.

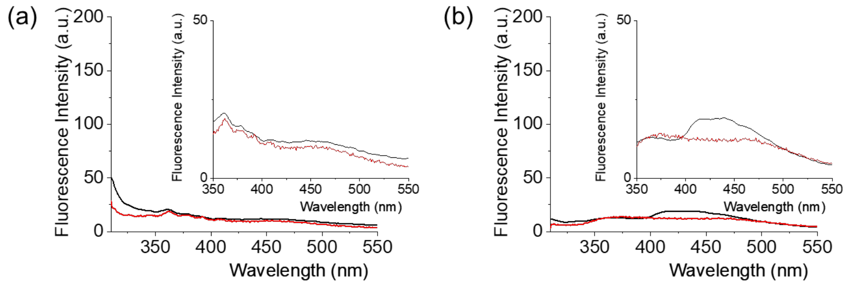

Figure S9. Emission spectra of solutions of **1** in (a) absence and (b) presence of 0.011 M of NaOH. In both cases, concentration of **1** is 1 mg/mL for the black data and 2 mg/mL for the red data. Insets show an expanded section of the corresponding graph. In all cases, solvent is 20:80 DMSO/water (v/v). The excitation wavelength is 300 nm.

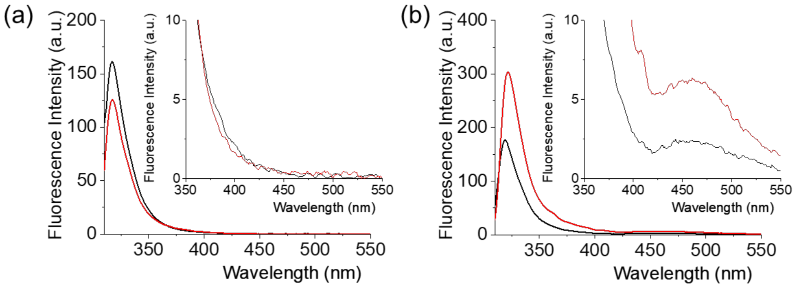

Figure S10. Emission spectra of solutions of **2** in (a) absence and (b) presence of 0.011 M of NaOH. In both cases, concentration of **2** is 1 mg/mL for the black data and 2 mg/mL for the red data. In all cases, solvent is 20:80 DMSO/water (v/v). Insets show an expanded section of the corresponding graph. The excitation wavelength is 300 nm.

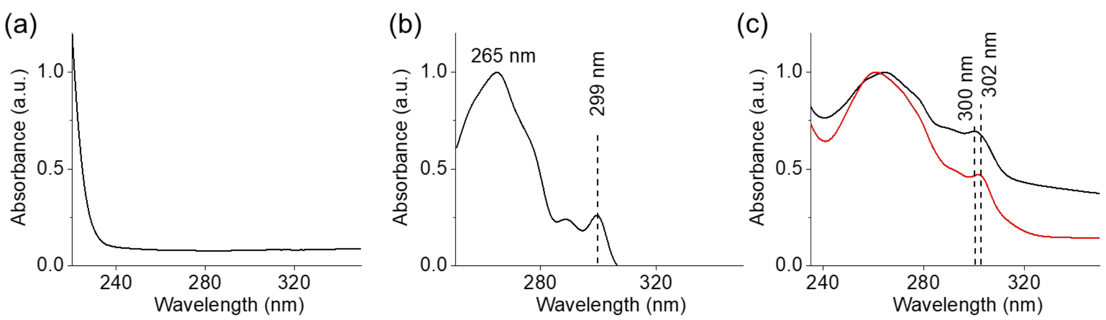

Figure S11. Normalized UV-vis spectra of (a) solution of **1**, (b) solution of **2**, and (c) the multicomponent gels of **1** and **2** obtained at pH 6.3 (black) and pH 9.9 (red). In all cases, initial concentrations of **1** and **2** are 2 mg/mL, solvent is 20:80 DMSO/water (v/v).

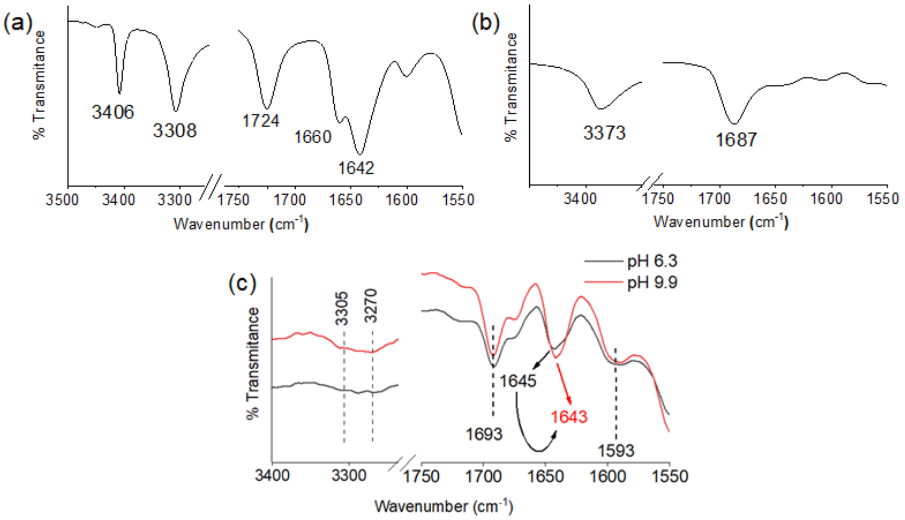

Figure S12. Partial FTIR spectra of (a) **1** and (b) **2** in their amorphous (solid) states. (c) Partial FTIR spectra of the multicomponent gels of **1** and **2** obtained from 20:80 DMSO/water (v/v) at pH 6.3 (black) and pH 9.9 (red).

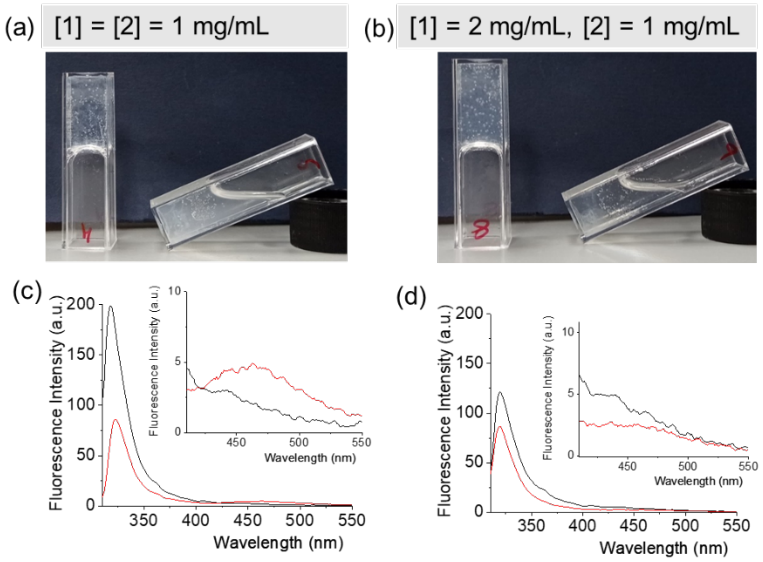

Figure S13. (a, b) Photographs of the gels and sols obtained from the mixtures of **1** and **2** at different concentrations of the components. In each photograph, the left cuvette represents the gel prepared from the mixture of **1** and **2** at a condition when **1** undergoes deprotonation but **2** exists as ammonium cation. The concentration of NaOH was (a) 0.0025 M and (b) 0.005 M. The right cuvette represents the phase change of the mixture of **1** and **2** at a condition when both the components undergo deprotonation. In all cases, the concentration of NaOH was 0.011 M. (c, d) Represent the emission profile for the gels and sols obtained from the study performed in figures (a, b) respectively. In all cases, the black data represent the emission spectra of the gels prepared at low pH. The red data represent the emission spectra of the sols obtained at high pH (0.011 M of NaOH). For (a-d), solvent is 20:80 DMSO/water (v/v).

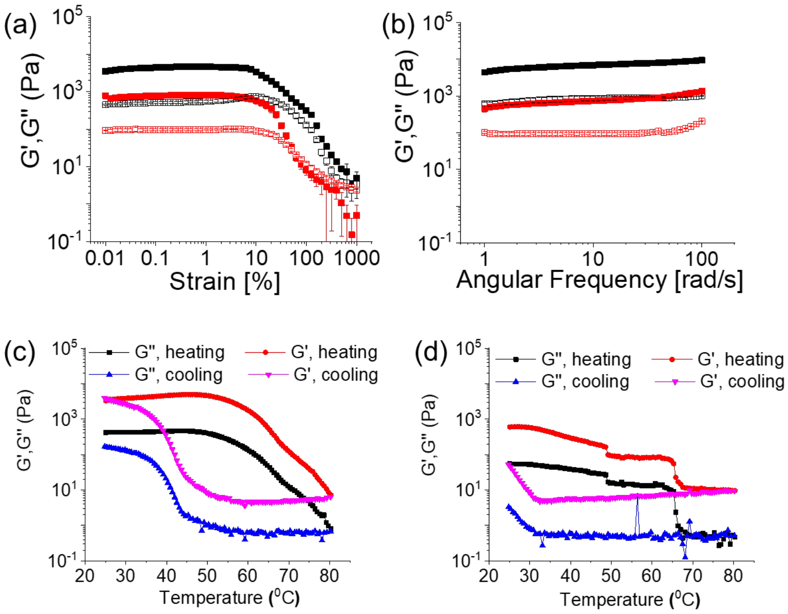

Figure S14. (a) Strain and (b) frequency sweep experiments of the multicomponent gel of **1** and **2** obtained at pH 6.3 (black) and pH 9.9 (red). The closed symbols represent G′, the open symbols G″. (c, d) Temperature sweep experiments of the multicomponent gel of **1** and **2** obtained at pH 6.3 (c) and pH 9.9 (d). In all cases, initial concentrations of **1** and **2** are 2 mg/mL, solvent is 20:80 DMSO/water (v/v).

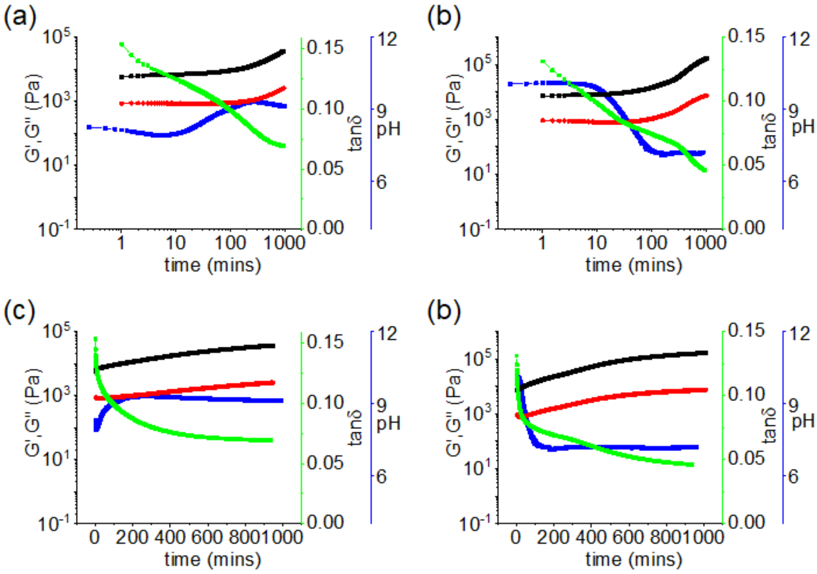

Figure S15. Variation of pH (blue), G′ (black), G″ (red) and tanδ (green) with time for the hydrogel of **1** and **2** upon addition of 0.006 M of NaOH at pH 6.3 (a) and 0.006 M of HCl at pH 9.9 (b). The final pH of the gels changes to pH 9.9 (a) and 6.3 (b). In both cases, initial concentrations of **1** and **2** are 2 mg/mL, solvent is DMSO/H_2O (20/80, v/v). Figures (c) and (d) represent Figure (a) and (b) using a linear X-scale, respectively. pH and rheology measurements were performed with two different vials under identical conditions.

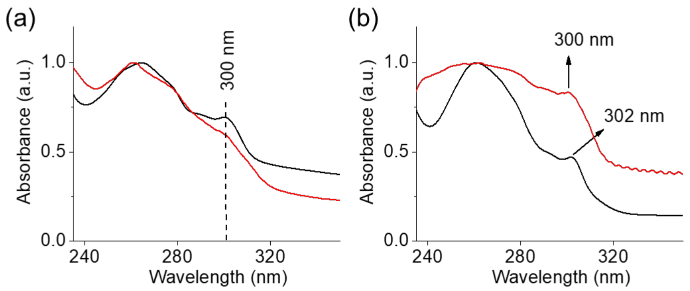

Figure S16. Normalized UV-vis spectra of the multicomponent gels of **1** and **2** before (black) and after (red) addition of 0.006 M of NaOH at pH 6.3 (a) and 0.006 M of HCl at pH 9.9 (b). The final pH of the gels (red data) changes to pH 9.9 (a) and 6.3 (b). In all cases, initial concentrations of **1** and **2** are 2 mg/mL, solvent is DMSO/H_2O (20/80, v/v).

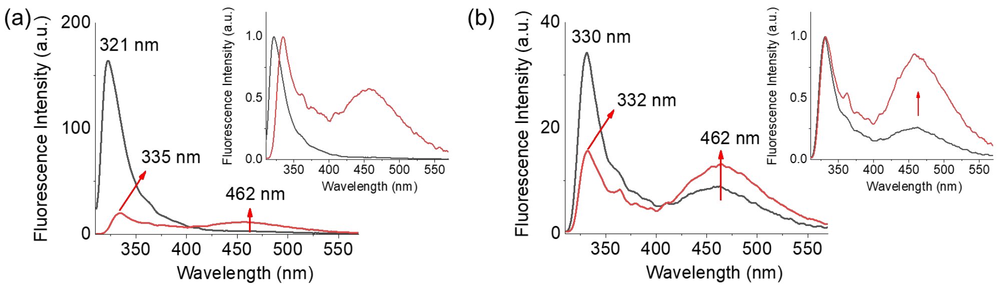

Figure S17. Emission spectra of the multicomponent gels of **1** and **2** at pH 6.3 (a) and pH 9.9 (b) prepared directly (black) and by PAF method (red data). Insets show the normalized graphs. In all cases, initial concentrations of **1** and **2** are 2 mg/mL, solvent is DMSO/H_2O (20/80, v/v).

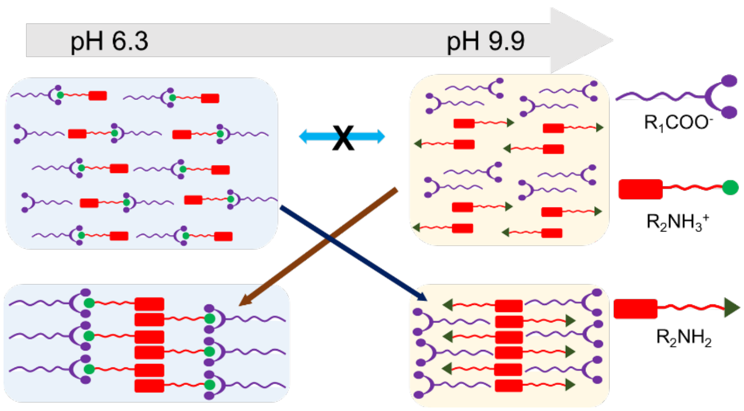

Figure S18. Cartoon representing the changes in molecular packing in the multicomponent gel of **1** and **2** during post assembly pH change.

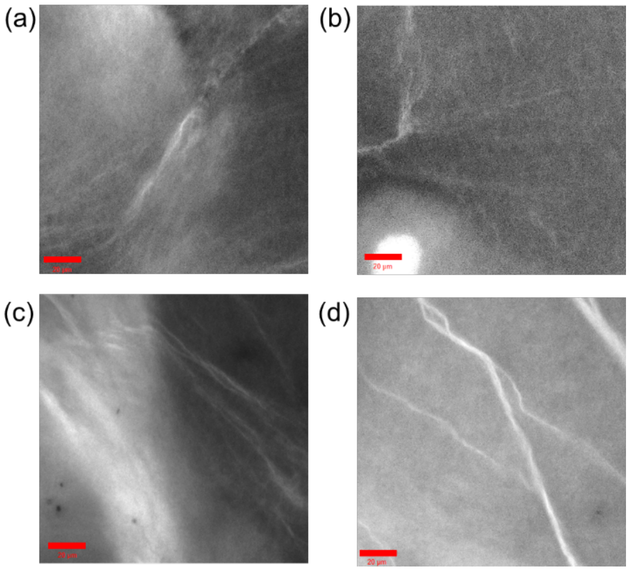

Figure S19. Confocal fluorescence microscopy images (scale bars represent 20 μm) of the multicomponent gel of **1** and **2** obtained at pH 9.9 (a, b) and pH 6.3 (c, d) involving post assembly pH change. In all cases, initial concentrations of **1** and **2** are 2 mg/mL, solvent is DMSO/H_2O (20/80, v/v).

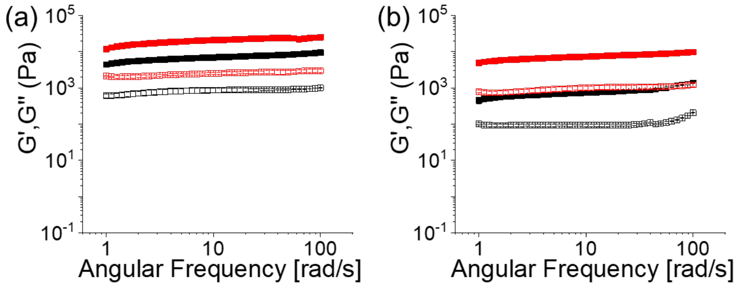

Figure S20. Frequency sweep experiments of the hydrogels of (**1**+**2**) at pH 6.3 (a) and pH 9.9 (b) prepared directly (black) and by PAF method (red). In all cases, initial concentrations of **1** and **2** are 2 mg/mL, solvent is DMSO/H2O (20/80, v/v). The closed symbols represent G′, the open symbols G″.

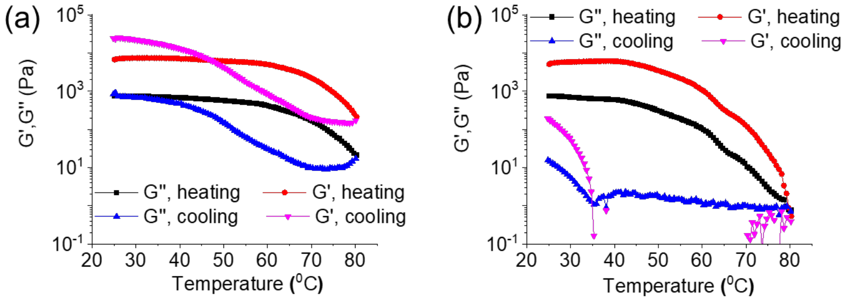

Figure S21. Temperature sweep experiments of the multicomponent gel of **1** and **2** obtained at pH 6.3 (a) and pH 9.9 (b) involving post assembly pH change. In both cases, initial concentrations of **1** and **2** are 2 mg/mL, solvent is 20:80 DMSO/water (v/v).

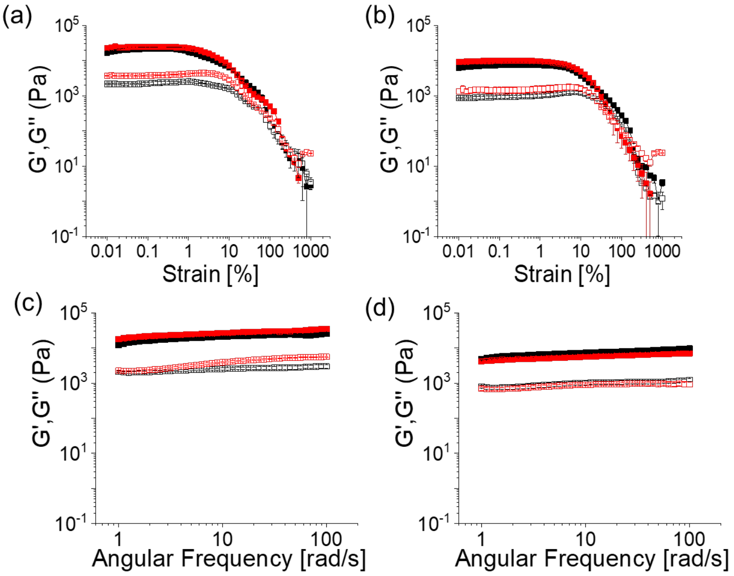

Figure S22. (a, b) Strain and (c, d) frequency sweep experiments of the hydrogels of (**1**+**2**) at pH 6.3 (a, c) and pH 9.9 (b, d) prepared under different conditions. In all cases, back data represent the gels prepared by PAF method and the red data represent the gels obtained after a pH cycle. In all cases, initial concentrations of **1** and **2** are 2 mg/mL, solvent is DMSO/H2O (20/80, v/v). The closed symbols represent G′, the open symbols G″.

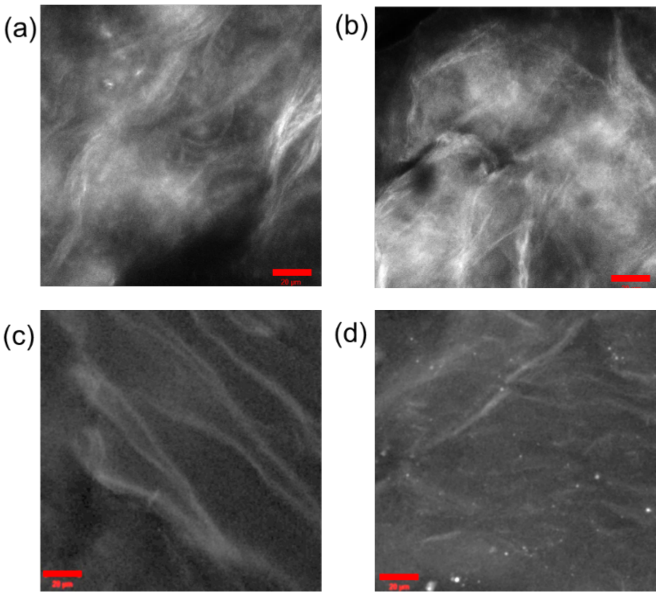

Figure S23. Confocal fluorescence microscopy images (scale bars represent 20 μm) of the multicomponent gel of **1** and **2** obtained at pH 6.3 (a, b) and pH 9.9 (c, d) after a pH cycle. In all cases, initial concentrations of **1** and **2** are 2 mg/mL, solvent is DMSO/H_2O (20/80, v/v).

References

1. J. K. Gupta, D. J. Adams and N. G. Berry, *Chemical Science*, 2016, **7**, 4713-4719.
2. www.sasview.org/
